# Water Behavior of Aerogels Obtained from Chemically Modified Potato Starches during Hydration

**DOI:** 10.3390/foods10112724

**Published:** 2021-11-07

**Authors:** Joanna Le Thanh-Blicharz, Jacek Lewandowicz, Zuzanna Małyszek, Przemysław Łukasz Kowalczewski, Katarzyna Walkowiak, Łukasz Masewicz, Hanna Maria Baranowska

**Affiliations:** 1 Department of Food Concentrates and Starch Products, Prof. Wacław Dąbrowski Institute of Agriculture and Food Biotechnology–State Research Institute, 40 Starołęcka St., 61-361 Poznań, Poland; zuzanna.malyszek@ibprs.pl (Z.M.); przemyslaw.kowalczewski@up.poznan.pl (P.Ł.K.); 2 Faculty of Engineering Management, Poznan University of Technology, 2 Jacka Rychlewskiego St., 60-965 Poznań, Poland; jacek.lewandowicz@put.poznan.pl; 3 Department of Food Technology of Plant Origin, Poznań University of Life Sciences, 31 Wojska Polskiego St, 60-624 Poznań, Poland; 4 Department of Physics and Biophysics, Faculty of Food Science and Nutrition, Poznań University of Life Sciences, 38/42 Wojska Polskiego St., 60-637 Poznań, Poland; katarzyna.walkowiak@up.poznan.pl (K.W.); lukasz.masewicz@up.poznan.pl (Ł.M.)

**Keywords:** ^1^H NMR, relaxation times, water activity, waxy starch

## Abstract

Aerogels are highly porous materials that are prepared by removing water held within a hydrogel in a manner that maintains the three-dimensional structure of the gel. Recently, there has been much interest in the preparation of aerogels from biopolymers, including starch. The applicability of native starches in the food industry is partially limited; therefore, the functional properties of starch are often improved by means of physical and/or chemical modification. The aim of the work was the analysis of molecular dynamics and the transport of water in aerogels obtained from native and chemically modified potato starches of the normal and waxy variety. Chemical modification with OSA (E 1450) as well as cross-linking with adipic anhydrite and acetylation (E 1422) had no significant impact on the hydration of potato starch aerogels as well as equilibrium water activity. The introduction of chemical moieties into starch macromolecules led to the improved binding of water by the biopolymer matrix; this was especially evident in the case of waxy starch derivatives. A increase in the amylopectin-to-amylose ratio of starch used for production of aerogels resulted in a decrease of equilibrium water activity along with spin-lattice relaxation time.

## 1. Introduction

The first aerogel was produced by Kistler in 1931 [1], using silica, gelatin, albumin, cellulose and agar. Aerogels are highly porous, ultralight materials that are prepared by removing water held within a hydrogel in a manner that maintains the three-dimensional structure of the gel [2]. The drying step of wet polysaccharide gel is crucial to maintain the integrity of the original three-dimensional structure. Drying can be accomplished through air-, freeze-, or supercritical fluid drying [3]. Recently, there has been much interest in the preparation of aerogels from biopolymers. Unlike inorganic and petroleum-derived compounds, the use of natural polysaccharides follows the trend of more sustainable, ecological and often non-toxic production. One of the most important polysaccharides used in various industries is starch, which is commonly used in the food industry, but also in the chemical and electrochemical industries [4,5,6]. One of the advantages of bio-aerogels over inorganic aerogels is their ability to undergo a functionalization by modification of hydroxyl groups of polysaccharide chains, which is especially effortless in the case of starch [7,8].

Native starches, due to their properties, pose various limitations in their industrial application. Therefore, various chemical modifications are carried out in order to improve functional properties of starch such as cold water solubility, viscosity and health-promoting properties [9,10,11,12,13,14]. In food production, many products are emulsion systems; therefore, modified starches with the ability to emulsify and stabilize the emulsion seem to be particularly important. Acetylated distarch adipate E 1422 is an esterified starch with anhydrides of adipic and acetic acids. It shows slight changes in viscosity, depending on the temperature, and is characterized by good resistance to changing thermal conditions but is weaker to mechanical forces. It gives a firm texture to products with a pH > 3.5. Used as a stabilizer, it allows maintaining of the physicochemical properties of products to which it is added, in small amounts, preventing or delaying spontaneous and unfavorable changes taking place [15,16]. On the other hand, octenyl succinic anhydride starch E 1450 (OSA starch), synthesized by an esterification reaction, stands out from the other chemically modified starches due to its high emulsifying ability and excellent interfacial properties [17]. This phenomenon is related to the presence of amphiphilic octenylsuccinic groups in the structure of this polysaccharide. This starch is able to efficiently decrease both surface and interfacial tension [18,19], and thus OSA starch shows wide application possibilities for creating films, coatings and gels, encapsulating other substances, and as an emulsifier [20]. The use of such modified starches for the production of aerogels makes it possible to obtain a product with significantly different sorption properties as well as physicochemical properties. To date, however, few papers have been published on the use of chemically modified starches in the production of aerogels [21,22]. It is therefore advisable to describe in detail the influence of the type of starch modification and the processing methods, such as the type of solvent or drying conditions, on the properties of aerogels [23].

Low-field nuclear magnetic resonance (LF NMR) spectroscopy is an excellent tool for analysis of water-biopolymer interactions [24,25,26,27]. It can be employed to study the physicochemical properties of bio-aerogels. The status of water molecules in biological systems depends on their interaction with biopolymers as well as with the low molecular mass components of the matrix [24,25,26]. Water activity is a parameter often used to describe this status. A detailed analysis of the changes in this parameter by reaching thermodynamic equilibrium in the biological systems makes possible to observe the molecular transport of water molecules in the biomaterial. This is important in aerogel technology development, as the sorption ability of aerogels is one of their most important features.

Therefore, the aim of the work was the analysis of molecular dynamics and the transport of water in aerogels obtained from native and chemically modified potato normal and waxy starches.

## 2. Materials and Methods

### 2.1. Materials

Normal potato starch, purchased from PPZ Trzemeszno (Trzemeszno, Poland), was characterized by an amylose and phosphorous content of 19.3% and 0.08%, respectively. Waxy potato starch “Eliane 100”, purchased from Avebe (Veendam, the Netherlands), had an amylose and phosphorous content of 3.0% and 0.06%, respectively [28]. Acetylated distarch adipate (E1422) from normal and waxy starches was prepared according to the method described by Luo et al. [29] and modified by Le Thanh-Blicharz et al. [30], with an approximate acetyl group content of 1.46% and adipic group content of 0.26% [9]. Sodium octenyl succinate starch (E1450) was obtained according to the method described by Jeon et al. [31], with an approximate octenylsuccinic group content of 2.25%.

### 2.2. Preparation of Aerogels

In order to obtain aerogels, a method was used in which water was successively displaced from the network structure of the gel by ethanol [32]. The aerogels were obtained by preparing 7% normal and waxy (also modified) potato starch suspension, kept at the boiling point in a water bath for 30 min. The pastes prepared in this way were poured into a flat container and, after cooling, placed in a refrigerator at 4 °C. After 24 h, the cooled, retrograded pastes were taken out and cut into cubes approximately 2 cm × 2 cm × 2 cm. The cubes were laid flat on flat surfaces and placed in a freezer at −15 °C for 24 h. The next day, the frozen cubes were removed from the freezer and allowed to thaw. Then, the cubes were drained of water and immersed in 500 mL of ethyl alcohol for 60 min. All of these operations were repeated three times. After the cubes were drained, the starch was dried at 50 °C in a circulating air oven until completely dry. Everything was ground with a ball mill.

Aerogels obtained from normal potato starch were denoted as ‘NPS’, from waxy starch ‘WPS’, from starches E 1422 and E 1450 for normal and waxy starches ‘E1422N’, ‘E1422W’, ‘E1450N’ and ‘E1450W’, respectively.

### 2.3. Aerogel Hydration

The hydration process was carried out at 20 °C with the use of ten solutions of sulfuric acid of various concentrations, prepared according to Ruegg [33], with different water activity ranging from 0.981 to 0.090 [34]. The hydration samples were spray-wetted to a moisture content of 12%, in order to standardize the initial water content. Approximately 1.5 g of aerogel in weighing pans were placed in desiccators above sulfuric acid solutions and weighed daily. Five samples were placed in each desiccator. One was used to observe the mass change and to determine the hydration value, two samples were used for NMR studies and two for water activity analysis. Hydration was carried out in a room with a controlled temperature of 20 °C. The process was carried out until the samples did not change their weight for two consecutive days. After hydration, all samples were transferred to NMR glass tubes and closed using Parafilm^®^. The water content in the samples of starch after hydration was determined by drying at 130 °C for 120 min. The hydration (h) was calculated on the basis of the masses of samples after hydration and drying, using the following formula [34]:

(1) $$h=\frac{m_{h}-{\;m}_{d}}{m_{d}}$$

where m_h_ and m_d_ are the weights after hydration and drying, respectively.

### 2.4. ^1^H NMR Relaxometry

The measurements of spin-lattice T_1_ relaxation times were performed at a controlled temperature of 20 °C. Measurements of relaxation times were made using MSL30 (WL Electronics, Poznań, Poland) operating at 30 MHz. The measuring head enables the analysis of samples with volumes from 0.1 to 0.2 cm^3^. To measure the relaxation time T_1_, a sequence of the inversion-recovery pulse sequence (π-TI-π/2) was used [35]. The interval between RF pulses (TI) varied from 1 to 100 ms. During each measurement, 32 FID signals were recorded. The repetition time for each measurement was 20 s. The values of spin-lattice relaxation times (T_1_) were calculated with the use of the CracSpin program [36], in which the Marquardt method enabled minimization by adjusting to the multi-exponential course of the magnetization regrowth curve. The increase in the longitudinal magnetization component (Mz) is described by the following formula:

(2) $$M_{z}\left(\mathrm{TI}\right)=M_{0}\left\{1-2p_{i}\sum_{i=1}^{n}e^{\frac{-\mathrm{TI}}{T_{1i}}}\right\}$$

where M_z_(t) is the actual magnetization value and M_0_ is the equilibrium magnetization value.

The spin-grouping method applied in the program made it possible to perform the two-dimensional analysis of FID signals in a time domain that made it possible to increase the accuracy of separation of the T_1_ relaxation time components.

### 2.5. Water Activity

Measurements of water activity were performed using the analyzer of water diffusion and activity ADA-7 (COBRABID, Poznań, Poland), with a system of automatic time recording of water evacuation runs from individual samples. Detailed characteristics of the experimental method were as described by Stangierski et al. [37], with a slight modification [38]. The starch powders were placed in measuring vessels with a diameter of 2 cm. The height of the sample was 1 cm. Samples were placed in the measuring chamber of the apparatus. Before the measurement, the chamber was dried to a water activity of 0.05. The temperature was stabilized at 20.0 ± 0.1 °C using Peltier modules. The duration of one measurement was set at 1000 s. The measurements of water activity were employed using following phenomenological model [39]:

(3) $$a_{w}\left(t\right)=\left(a_{0}+a_{p}\right)e^{-V_{D^{t}}}+\left(a_{p}-a_{r}\right)e^{-V_{p^{t}}}$$

where *a_w_(t)* is the momentary/temporary water activity, *a_0_* is the initial water activity, *a_p_* is the border water activity (indirect), *a_r_* is the water activity in a state of balance (final), *V_D_* is the transport speed/velocity and *V_p_* is the speed of surface conductance.

### 2.6. Statistical Analysis

Statistical analysis of the data was performed with Statistica 13 (Dell Software Inc., Round Rock, TX, USA) software. All measurements were studied using one-way analysis of variance, independently for each dependent variable. Post hoc Tukey honest significant difference (HSD) multiple comparison tests were used to identify statistically homogeneous subsets at α = 0.05. Principal component analysis (PCA) was performed using selected data obtained in the analyses.

## 3. Results and Discussion

**Figure 1:**
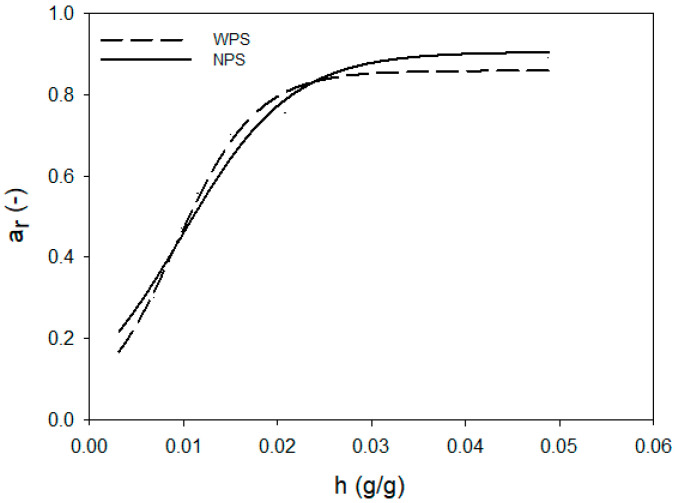
Changes in the equilibrium value of water activity during hydration of native potato starch aerogels.

**Figure 2:**
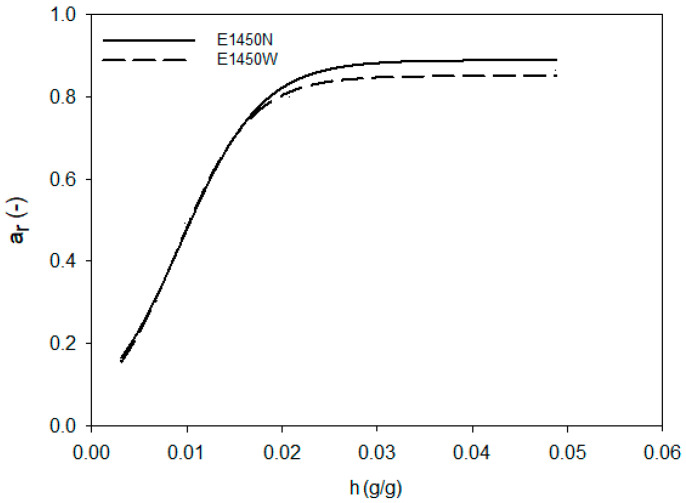
Changes in the equilibrium value of water activity during hydration of OSA starch aerogels.

**Figure 3:**
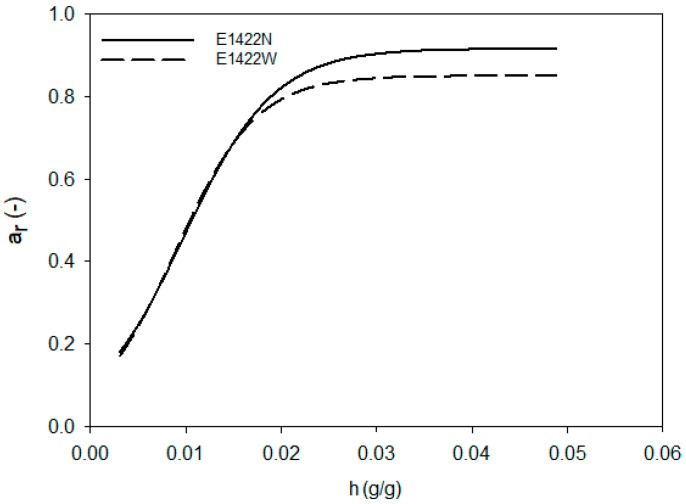
Changes in the equilibrium value of water activity during hydration of acetylated distarch adipate aerogels.

Aerogels, in terms of their affinity to water, resemble other food powders, such as powdered milk or cocoa. The properties of water in such hydrated systems are mainly observed by studying the activity of water. The changes in a_w_ in such relatively dry systems are described by sigmoid curves [40,41]. Water activity tests were carried out in aerogels with a controlled amount of hydration water. Figure 1, Figure 2 and Figure 3 show changes in the equilibrium value of water activity (a_r_) in starch aerogels. The type of chemical modification of starch performed did not affect the a_r_ of aerogels, regardless of the relative humidity in which they were conditioned. On the other hand, significant changes were observed between aerogels obtained from normal and waxy preparations. At initial stage of the hydration the process progressed in a similar manner for all investigated samples. As the value of hydration water got closer to 0.02 g per gram of starch aerogel, differences between values of equilibrium water activity were observed. Aerogels derived from normal preparations were characterized by significantly higher plateau a_r_ that waxy ones. The observed phenomena should be linked with the presence of amylose, but the physics behind that process cannot be solely explained by the a_r_ analysis. Waxy starches are known to be composed entirely of amylopectin [42], whereas normal potato starch contains approximately 20–30% of amylose [43,44]. Amylopectin is the branched fraction of starch with a molecular mass higher by over one level of magnitude [45]. The molecular differences between those two fractions may partially influence the binding of water in starch aerogels. In particular, it is important to take into consideration that the degree of branching of normal and waxy potato starches is comparable [28]. Moreover, amylose is the fraction believed to be more susceptible to rapid retrogradation [46], which leads to reformation of the crystalline structure after starch gelatinization. The preparation of starch aerogels consists of the repeated freezing and thawing of starch pastes, thus providing perfect conditions for retrogradation [47]. Therefore, the formation of retrograded starch crystallites could partially influence the observed phenomenon of a higher a_r_ plateau of aerogels as derived from the normal potato starch variety. Moreover, the effect of frost damage, which could possibly affect the waxy variety to a higher extent due to its larger molecular weight [28], should not be overlooked [48].

The analysis of Figure 1, Figure 2 and Figure 3 indicated that the changes of a_r_ during the hydration of starch aerogels are described by the sigmoidal function (R^2^ > 0.997), in the form of

(4) $$a_{r}=\frac{A}{1+\exp\left(\frac{-h-C}{B}\right)}$$

where:

A—maximum water activity reached by aerogel,
B—lowest hydration value at maximum water activity (inflection point),
C—limit hydration level at which only bound water is present in the system,
h—the hydration value.

Table 1 summarizes the values of the function parameters (Equation (4)) for individual types of starch aerogels. The presented data show that significant differences were only present between the A parameter of the equation, which indicates maximum water activity that the aerogel can reach. The aforementioned parameter had a lower value for aerogels obtained from waxy starch preparations, which was related to a lower value of plateau a_r_, shown in Figure 1, Figure 2 and Figure 3. The remaining parameters of the sigmoidal function (B, C) did not differentiate the investigated preparations to a significant extent; however, higher values of both B and C were observed for the native preparation. This confirms that, below the values of the hydration increase of 0.02 g/g, the water activity of all starch preparations was similar. At higher hydration levels, when free water started to be present in the system, differences between waxy and normal starches could be observed. This, apart from the previously mentioned observations, could be also partially linked to the phosphorous content of the samples, as other water properties such as swelling power and solubility are strongly correlated with it [49]. 

The analysis of Equation (3) also proved that type of employed chemical modification (E1422 and E1450) had no significant impact on maximal equilibrium water activity as well as amount of water that can be bound to the starch macromolecule. This indicates that food grade starch preparations are characterized by similar water behavior, regardless of the degree of substitution of the modifying group content as recommended by the FAO/WHO, i.e., <0.135% for adipic, <2.5% for acetyl, and 3% for octenylsuccinyl [50].

Starch hydration under controlled conditions made it possible to obtain research material with different water organization. However, water activity measurements do not allow for a precise evaluation of the differences between the binding of water in materials such as aerogels. The observation of changes in a_r_ may be useful when designing food products, but the obtained results provide information regarding water behavior only on a macroscopic scale. Therefore, in order to understand this phenomenon, it is necessary to use research methods that allow for the analysis of water binding at a molecular level.

Molecular properties of water in biopolymer systems can be thoroughly analyzed using LF NMR [51,52,53]. This method consists of the determination of the spin-lattice relaxation time T_1_ and the spin-spin relaxation time T_2_, which describe the transfer of previously absorbed energy from spin to the surrounding environment and from spin to neighboring spins, respectively. In solid-state systems, the protons of water molecules have limited mobility; therefore, a decrease of value of the relaxation times is observed with an increase in the amount of water molecules bound to the biopolymer matrix by hydrogen or ionic bonding [54]. Research on hydrated cellulose materials shows that two components of spin-lattice relaxation times are observed when the amount of water is greater than that which saturates all sorption sites [55]; this phenomenon was also shown for modified starches in their granular form [56].

**Figure 4:**
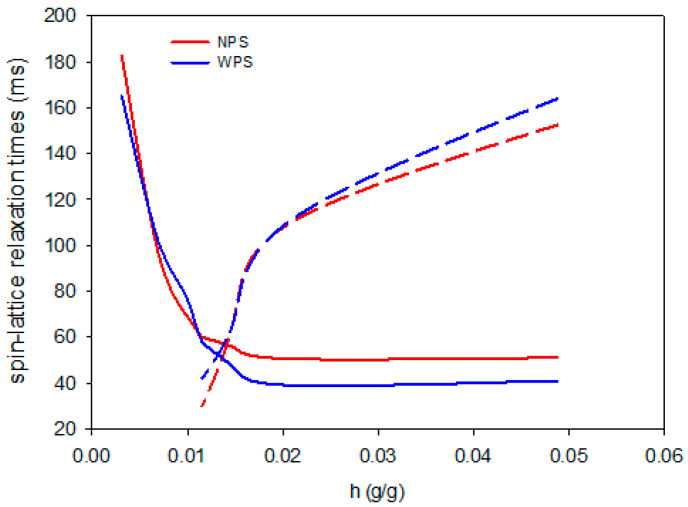
Changes in the short T_11_ (solid) and long T_12_ (dashed) components of spin-lattice relaxation times during hydration of native potato starch aerogels.

**Figure 5:**
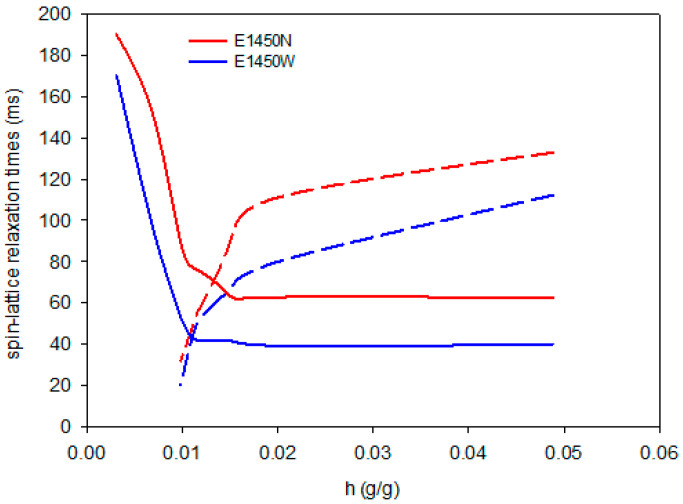
Changes in the short T_11_ (solid) and long T_12_ (dashed) components of spin-lattice relaxation times during hydration of OSA starch aerogels.

**Figure 6:**
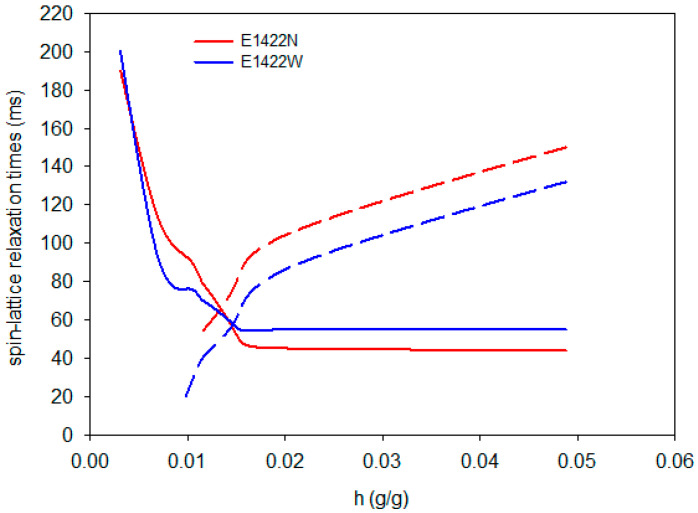
Changes in the short T_11_ (solid) and long T_12_ (dashed) components of spin-lattice relaxation times during hydration of acetylated distarch adipate aerogels.

Figure 4, Figure 5 and Figure 6 show changes in the values of spin-lattice relaxation times depending on the hydration water content in the tested samples. At relatively low increases in water content, i.e., below 0.01 g/g, only one component of spin-lattice relaxation time was observed. As the hydration process proceeded, the T_1_ shortened, thus indicating more sites where water was bound to the biopolymer. Above a hydration value of 0.01 g/g, two components of T_1_ emerged, namely short T_11_ and long T_12_. At this point, all sorption sites of the aerogel were fully saturated, and T_11_ values started to reached their plateau. The long components of spin-lattice relaxation time increased rapidly from that point to a level of hydration of 0.02 g/g, whereupon linear increase could be observed up to 0.05 g/g. The previously described process of changes of spin-lattice relaxation time during hydration of starch aerogels was observed for all investigated samples, regardless of starch variety or type of modification. Aerogels derived from native starches were characterized by similar and relatively smooth relaxation curves with one prolonged inflection point at a hydration value between 0.01–0.02. In the case of OSA aerogels, much sharper inflections of the curves were observed. Acetylated distarch adipate aerogels were characterized by the most prolonged inflection. This indicates that while the absolute value of hydration at which free water starts to be present in the system is comparable for all preparations, the binding of water is affected by type of chemical modification performed.

The analysis of the water molecular dynamics results of the aerogels showed the presence of two fractions of water, which are described by the different functions presented below.

(5) $$T_{11}=f+g\;\exp\left(\frac{-h}{k}\right)$$

(6) $$T_{12}=m+\frac{p}{1+\left(\frac{h-s}{w}\right)}$$

Using the above equations, the hydration point values at which all sorption sites of the aerogel are fully saturated (h_0_) were calculated (using the fitting parameters presented in Supplementary Tables S1 and S2). Moreover, the values of the spin-lattice relaxation times T_1_ corresponding to the hydration value of h_0_ were also determined. The aforementioned molecular saturation parameter was compared with the macroscopic one (h_ar_), calculated on the assumption that above full saturation of the aerogel, the a_r_ reaches its plateau. Similarly to h_0,_ the spin-lattice relaxation times (T_11ar_ and T_12ar_) corresponding to the hydration value of h_ar_ were determined. Table 2 shows the values of the compared parameters.

The hydration level values that corresponded to the saturation of all sorption sites derived both from a_r_ and T_1_ measurements differed insignificantly when compared between samples. This indicates that both the variety as well as the type of chemical modification of the starch do not influence the sorption capacity of aerogel. Presumably, the physical modification of starch that leads to the formation of the aerogels’ porous structure is the factor determining this property. On the other hand, h_ar_ values were approximately twice as high as h_0_, indicating that measurement at the molecular level is significantly more accurate. The h_ar_ represented the hydration value at which a linear increase of T_12_ relaxation time was observed.

The T_1_ relaxation time for water, which saturates all sorption sites, depends on the starch from which the aerogel was obtained. It was found that for systems obtained from waxy starches, the relaxation times are shorter than for starches with higher amylose content. Moreover, the type of chemical modification employed resulted in a decreased relaxation rate, which was especially evident in the case of waxy starch aerogels. Although chemical modification and amylose/amylopectin ratio do not influence the amount of hydration water absorbed by the aerogel, they enhance the binding of water to the polymer matrix. Similar observations were made for T_11ar_, but the differences were less evident. This corresponds well with the analysis of the parameters describing the functions of changes in the equilibrium water activity; however, once again it should be emphasized that the differences between T_1_ of aerogels measured at h_0_ are more pronounced. 

**Figure 7:**
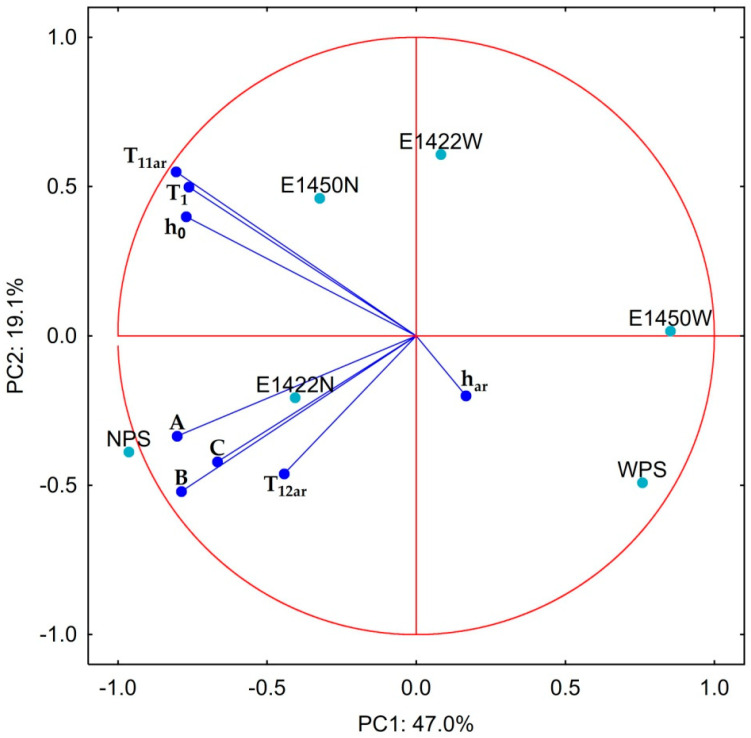
Principal component analysis biplot of the data characterizing the dependence of the equilibrium water activity as a function of hydration (A, B, C) and related to the hydration point at which all sorption sites of the aerogel are fully saturated (h_0_, h_ar_, T_1_, T_11ar_, T1_2ar_). NPS: aerogels obtained from normal potato starch; WPS: from waxy starch; E 1422N, E 1422W, E 1450N and E 1450W: from E 1422 and E 1450 for normal and waxy starches, respectively.

In order to correlate and systematize parameters describing the water properties of modified starch aerogels, principal component analysis was performed. PCA biplot presents two first principal components that explain almost two-thirds of total variance between samples (Figure 7). The first component represents mostly the variation that results from the amylose content of the starch used for aerogel production. The second component is partially determined by the chemical modification of starch used. Loading values can be separated into two subsets. The first one groups parameters related to water, which saturates all sorption sites. The second includes parameters characterizing the dependence of the equilibrium water activity as a function of hydration and long component of the spin-lattice relaxation time. Observed similarities were indicated previously for both of these groups. These observations indicate that measurement of relaxation rates at a point in which free water starts to be present in the system may be sufficient for characterization of water behavior in starch aerogels. 

## 4. Conclusions

Chemical modification with OSA (E 1450) as well as cross-linking with adipic anhydrite and acetylation (E 1422) had no significant impact on hydration of potato starch aerogels. However, the introduction of chemical moieties into starch macromolecules improved the binding of water by the biopolymer matrix, especially in case of waxy starch derivatives. Nevertheless choosing the normal over waxy potato starch variety is preferred during production of aerogels with stronger water-biopolymer interactions.

Moreover it was stated that analysis of water behavior at the macroscopic level (water activity) is insufficient for characterization of modified starch aerogels. It is crucial to extended the analysis to determination of molecular dynamics in order to fully characterize water behavior in starch aerogels.

## Figures and Tables

**Table 1:** Parameters characterizing the dependence of the equilibrium water activity as a function of hydration.

Scheme	A	B	C
NPS	0.904 ± 0.002	0.006 ± 0.001 ^a^	0.010 ± 0.001 ^a^
WPS	0.858 ± 0.001 ^a^	0.004 ± 0.001 ^a^	0.009 ± 0.001 ^a^
E1450N	0.888 ± 0.003	0.004 ± 0.001 ^a^	0.009 ± 0.001 ^a^
E1450W	0.858 ± 0.002 ^a^	0.004 ± 0.001 ^a^	0.009 ± 0.001 ^a^
E1422N	0.916 ± 0.001	0.005 ± 0.001 ^a^	0.009 ± 0.001 ^a^
E1422W	0.850 ± 0.002	0.004 ± 0.001 ^a^	0.009 ± 0.001 ^a^

NPS: aerogels obtained from normal potato starch; WPS: from waxy starch; E 1422N, E 1422W, E 1450N and E 1450W: from E 1422 and E 1450 for normal and waxy starches, respectively. Mean values with the same letters (^a^) in the columns are not significantly different at α = 0.05.

**Table 2:** Hydration values saturating all sorption sites in the aerogel and corresponding to their relaxation times.

Sample	T_1_ (ms)	h_0_ (g/g)	h_ar_ (g/g)	T_11ar_ (ms)	T_12ar_ (ms)
NPS	57.5 ± 0.4 ^a^	0.014 ± 0.002 ^a^	0.021 ± 0.001 ^ab^	56.8 ± 0.5 ^b^	111.1 ± 0.5
WPS	30.3 ± 0.1	0.012 ± 0.005 ^a^	0.024 ± 0.002 ^ab^	38.9 ± 0.7 ^a^	119.1 ± 0.3
E1450N	71.4 ± 0.5	0.013 ± 0.001 ^a^	0.020 ± 0.002 ^a^	59.9 ± 0.7	113.2 ± 0.2
E1450W	43.2 ± 0.5	0.011 ± 0.003 ^a^	0.021 ± 0.001 ^ab^	38.6 ± 0.5 ^a^	81.5 ± 0.7
E1422N	66.4 ± 0.3	0.013 ± 0.003 ^a^	0.025 ± 0.002 ^b^	49.6 ± 0.7	116.7 ± 0.6
E1422W	56.5 ± 0.2 ^a^	0.015 ± 0.002 ^a^	0.024 ± 0.001 ^ab^	57.4 ± 0.5 ^b^	92.8 ± 0.3

NPS: aerogels obtained from normal potato starch; WPS: from waxy starch; E 1422N, E 1422W, E 1450N and E 1450W: from E 1422 and E 1450 for normal and waxy starches, respectively. Mean values with the same letters (^a,b^) in the columns are not significantly different at α = 0.05.

## Data Availability

All data generated or analyzed during this study are included in this published article.

## Supplementary Materials

The following are available online at https://www.mdpi.com/article/10.3390/foods10112724/s1, Table S1: Fitting parameters for equation #4, Table S2: Fitting parameters for equation #5.
