# Supplementary material for: Water Behavior of Aerogels Obtained from Chemically Modified Potato Starches during Hydration
Source: Foods. 2021 Nov 7;10(11):2724. doi: 10.3390/foods10112724 (PMC8623613; doi:10.3390/foods10112724)
Supplement: Supplementary file 1 [file foods-10-02724-s001.zip › foods-1414919-supplementary.pdf]

Table S1. Fitting parameters for equation #4

sample	f	g	k
NPS	155±7	-448±11	0.009±0.002
NPW	174±3	-298±10	0.018±0.005
E1450	134±5	-359±8	0.007±0.003
E1450W	114±2	-226±10	0.018±0.004
E1422	154±7	-295±9	0.011±0.002
E1422W	140±2	-226±7	0.014±0.001

Table S2. Fitting parameters for equation #5

sample	m	p	s	w
NPS	50±3	175±10	0.005±0.001	-0.0018±0.0001
NPW	39±2	198±12	0.005±0.001	-0.0029±0.0002
E1450N	63±4	127±9	0.007±0.001	-0.0007±0.0001
E1450W	40±2	202±7	0.004±0.001	-0.0018±0.0003
E1422	41±4	299±10	0.002±0.001	-0.0046±0.0001
E1422W	53±6	208±9	0.004±0.001	-0.0029±0.0002
